# Ascending Colon Volvulus: The Enigma of Double Gastric Bubble

**DOI:** 10.1155/cris/9531608

**Published:** 2025-04-17

**Authors:** Paschalis Gavriilidis, Pantelis Xanthakos

**Affiliations:** Department of Surgery, General Hospital of Syros, “Vardakeio and Proïo”, Hermoupolis, Syros 84100, Greece

**Keywords:** ascending colon, caecum, volvulus

## Abstract

**Introduction:** Volvulus of the caecum, ascending colon and first third of the transverse colon is a very rare surgical emergency. Timely diagnosis and intervention can avert serious complications.

**Case Report:** A 54-year-old woman presented to the emergency department with colicky epigastric pain radiating to the left iliac fossa that lasted for 12 h. Vital signs were stable upon arrival at the hospital. Physical examination revealed that left abdomen and suprapubic fullness and bowel sounds were recorded, and rebound tests were negative. Laboratory results were neutrophils: 78% (35%–72%), lymphocytes: 16% (20%–45%), HB: 11 g/dL (12–16), HCT: 33% (36%–48%); all the rest were normal. Furthermore, computed tomography revealed a distended close of the large bowel extending from the left hypochondrium to the left iliac fossa. During explorative laparotomy, volvulus of the first third of the transverse, ascending colon and caecum was detected. Because the viability of the bowel wall was compromised, right extended hemicolectomy was performed with consequent ileotransverse anastomosis. The postoperative period was uneventful, and the patient was discharged on the fourth postoperative day.

**Conclusions:** Expeditious diagnosis and early intervention of very rare surgical emergencies such as ascending colon volvulus may avert disastrous complications.

## 1. Introduction

Volvulus of the ascending colon and caecum is a very rare surgical emergency and comprises only 1.15% of all mechanical bowel obstructions, excluding those resulting from hernia obstruction [1]. Any segment of the large bowel may rotate if its mesentery is long and wide and narrows at the base; in particular, volvulus of the sigmoid comprises 60%–80% of all colonic volvulus cases; cecal volvulus accounts for 20%–40%, volvulus of the transverse colon 3% and splenic flexure only 2% of cases [1–4]. Volvulus of the ascending colon may arise from persistent ascending mesocolon; it is a rare embryological anomaly that occurs when the primitive dorsal mesocolon fails to fuse with the parietal peritoneum during the fifth month of gestation [5]. Because of its rarity, diagnosis and intervention are often delayed and thus, associated with serious complications [6, 7].

## 2. Case Report

A woman in her fifties presented to the emergency department of the rural hospital with colicky epigastric pain radiating to the left iliac fossa that lasted for 12 h. Vital signs were stable upon arrival at the hospital. Physical examination revealed left abdomen fullness, mild epigastric pain and pain in the left abdomen. Giordano, Murphy and rebound tests were negative. Bowel sounds were also recorded. The patient reported episodes of suprapubic fullness. Moreover, she reported that she passes gasses and stools regularly.

Her medical history included gastroesophageal reflux disease, Hashimoto disease and dyslipidemia.

Laboratory results were WBC: 8.09 K/*µ*L (4–11), neutrophils: 78% (35%–72%), lymphocytes: 15.8% (20%–45%), Hb: 11 g/dL (12–16), HCT: 33% (36%–48%); all the rest were normal. Patient never had any past operations.

**Figure 1:**
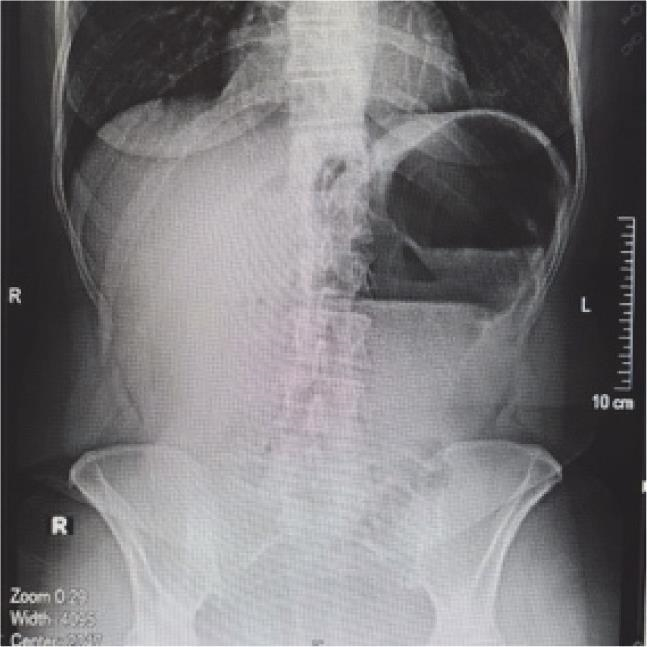
Double gastric bubble. The front bubble is the dilated stomach and the one behind the caecum.

**Figure 2:**
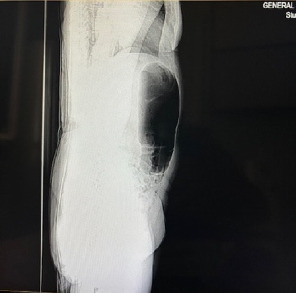
Closed loop of the ascending colon extending from the left hypochondrium to the left iliac fossa.

**Figure 3:**
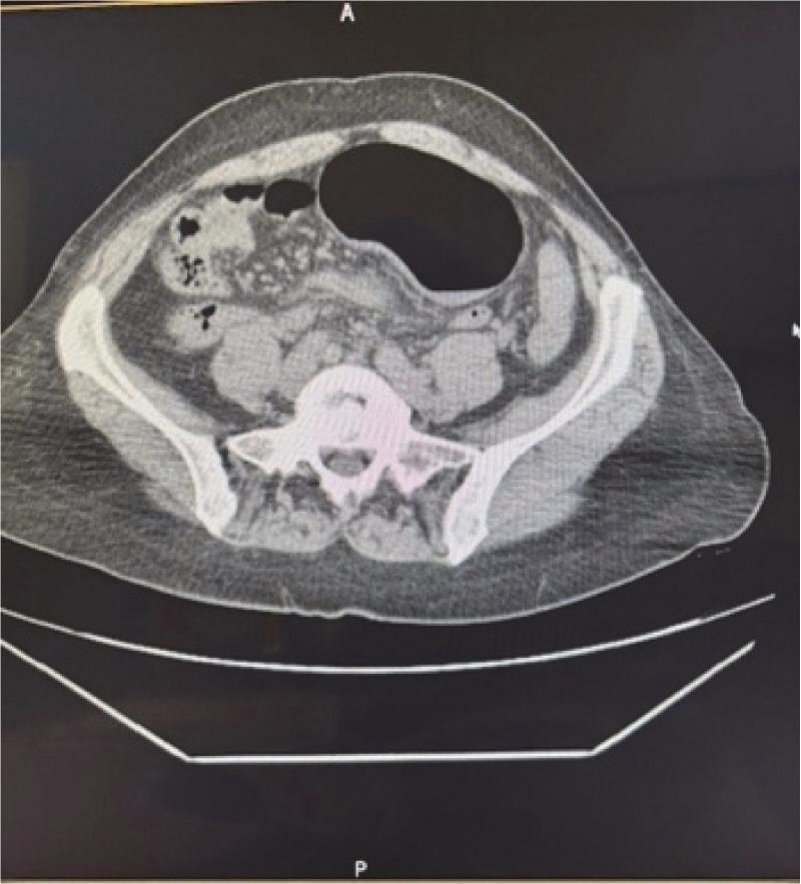
Coffee bean sign of the caecum at the level of the left iliac fossa.

Plain abdominal radiography revealed characteristic signs of a double gastric bubble (Figure 1) [8]. Computed tomography (CT) revealed a distended closed loop of the large bowel extending from the left hypochondrium to the left iliac fossa, and a coffee bean sign, the characteristic feature of the twisted caecum (Figures 2 and 3).

**Figure 4:**
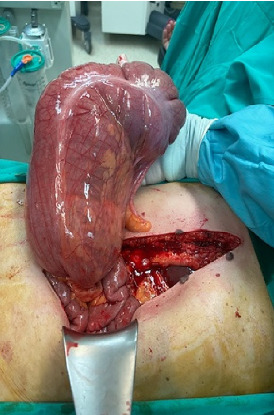
Twisted first part of the transverse, ascending colon and caecum located on the left hypochondrium.

During explorative laparotomy, volvulus of the first third of the transverse, ascending colon and caecum was detected (Figure 4). Because the viability of the bowel wall was compromised, right extended hemicolectomy was performed with consequent ileotransverse anastomosis. The post-operative period was uneventful, and the patient was discharged on the fourth post-operative day.

## 3. Discussion

It has been reported that the volvulus of the colon is always due to a congenital lack of fusion and fixation of the caecum and the ascending colon to the parietal peritoneum. Of note, in the fifth month of gestation, during the third stage of intestinal rotation, a process of fusion takes place between the parietal peritoneum and the mesentery of the ascending colon [5].

The main cardinal abdominal findings consist of left abdominal distention, generalised tenderness and spasm of the abdominal musculature. In the early cases, on auscultation, peristalsis is obstructive in character and absent in late cases. The laboratory findings are not remarkable [1].

Imaging findings of the abnormal position of the caecum in the left hypochondrium and twisting mucosal pattern help essentially to differentiate the volvulus from the obstructing tumours [1].

Of note, the position of the gas-filled caecum in the left hypochondrium may produce the image of the double gastric bubble. Young et al. [1] reported that some authors resolved this problem either by intubating the stomach or outlining it with contrast and distinguishing it from the caecum (Figure 1) [1]. Of note, the characteristic image of the twisted caecum is the coffee bean sign placed on the left abdomen and its hilum oriented to the right (Figure 3) [1].

The treatment of choice of the volvulus of the ascending colon is surgical. However, there is no set of rules that supersedes surgical judgment. For the cases that the blood supply has been comprised for longer usually more radical surgery is needed [1]. In the cases that the bowel wall is healthy, the volvulus must be reduced and the twisted part of the colon should be fixated on the parietal peritoneum to prevent recurrence. Furthermore, when there is the slightest question of the competency of the wall of the ascending colon, the surgeon should proceed by applying the method of Mikulicz [2]. A Mikulitcz procedure in the context of an obstructed colon refers to creation of a temporary double-barrel colostomy to relieve the obstruction, before any definitive operation [2].

## 4. Conclusions

Up to the authors best knowledge, this is the first case of simultaneous volvulus of the first third of the transverse, ascending colon and caecum. Presentation on the plain abdominal film of the sign of the double gastric bubble and on the CT scan distended closed loop of the large bowel may raise suspicion of the diagnosis of the volvulus of the ascending colon. Early intervention may prevent serious complications.

## Data Availability

The data that support the findings of this study are available on request from the corresponding author. The data are not publicly available due to privacy or ethical restrictions.
